# Risk Factors and Patient Outcomes Associated With Immediate Post-operative Anasarca Following Major Abdominal Surgeries: A Prospective Observational Study From 2019 to 2021

**DOI:** 10.7759/cureus.20631

**Published:** 2021-12-23

**Authors:** Satya P Meena, Metlapalli V Sairam, Ashok K Puranik, Mayank Badkur, Naveen Sharma, Mahendra Lodha, Mahaveer S Rohda, Nikhil Kothari

**Affiliations:** 1 General Surgery, All India Institute of Medical Sciences, Jodhpur, IND; 2 Anesthesiology and Critical Care, All India Institute of Medical Sciences, Jodhpur, IND

**Keywords:** mortality, laparotomy, clavien-dindo grading, edema, albumin, anasarca

## Abstract

Introduction: Anasarca is well-known and refers to generalized edema caused by underlying clinical conditions and unknown risk factors in the patient. However, it is a relatively unexplored postoperative symptom following major abdominal surgeries. It is associated with poor patient outcomes in terms of delayed recovery and associated severe complications. Pedal edema is an early sign of post-operative anasarca, which progresses into an unfavorable clinical condition due to generalized edema followed by multiple organ dysfunction.

Aim: This study aimed to assess risk factors and complications associated with postoperative anasarca among patients undergoing major abdominal surgery.

Methods and material: The prospective observational study included 241 patients undergoing major abdominal surgeries from July 2019 to February 2021 in a tertiary care health centre in Rajasthan, India. Risk factors like age, nutritional parameters, addictions like smoking, alcohol intake, opium intake, leukocytosis, and Charlson Comorbidity Index were assessed. Postoperative complications were graded by the Clavien-Dindo grading system. Mean, standard deviation, percentages, Pearson’s Chi-square test and Student’s t-test were used to analyze the data.

Results: The incidence of anasarca was found to be 29.87%. Nutritional risk screening (NRS) 2002 score, albumin, age > 60 years and raised leukocyte counts were found to significantly correlate (p-value <0.05) with the development of anasarca postoperatively. Postoperative complications, according to Clavien-Dindo grading, were 16.67% in grade I (p value=0.002), 13.89% in grade II (p-value =0.199), 1.39% in grade III (p value=0.049), 20.83% in grade IV (p value<0.001), and 41.67% in grade V (p value<0.001).

Conclusion: Higher NRS 2002 score, low albumin levels, age > 60 years and raised leukocyte counts are significantly correlated with the development of postoperative anasarca. Postoperative anasarca is found to be a significant predictor of poor prognosis of patients undergoing major abdominal surgery.

## Introduction

Edema is defined as a clinically apparent increase in the interstitial fluid volume, which develops when Starling forces are altered so that there is an increased flow of fluid from the vascular system into the interstitium. Anasarca refers to gross generalized edema [1]. Hypoalbuminemia may not generate generalized edema by itself and is frequently linked to a trigger factor or other comorbidities. Fluid accumulation has been linked to a slower gastrointestinal recovery, slower wound healing, infective problems, sepsis, and a longer hospital stay [2]. Previous works of literature have shown increased morbidity associated with postoperative edema following extra-abdominal surgery like plastic, maxilo-facial and orthopedic surgery. However, there are lacunae in the literature for risk factors associated with postoperative anasarca and related morbidity following abdominal surgery. This study aimed to assess postoperative anasarca, its incidence, risk factor and outcome of patients after major abdominal surgeries.

## Materials and methods

The study was a hospital-based prospective observational study and was conducted upon 241 adult patients undergoing major abdominal surgery. The study has been conducted from July 2019 to February 2021 in the Department of General Surgery at a tertiary care health centre in Rajasthan, India. 

The study included all the patients of age more than 18 years and who underwent major abdominal surgery in the terms of minimum 10 cm size midline laparotomy. It excluded all patients who had preoperative anasarca and postoperative edema due to drug reaction, vascular cause, cellulitis or lymphangitis. 

The preoperative nutritional status of the patients was assessed by parameters such as the Nutritional Risk Screening (NRS) 2002 score (Table 1), blood parameters like hemoglobin, albumin levels, anthropometric parameters for example mid-arm circumference (MAC), mid-thigh circumference (MTC) and body mass index (BMI). Several risk factors for the development of postoperative anasarca were evaluated including age, addictions for smoking, alcohol, opium, blood parameters such as raised leukocyte counts (>11 x 10³/mm3) and comorbidities graded by the Charlson comorbidity index (CCI).

**Table 1:** NRS 2002 for the hospitalized patients NRS- Nutritional risk screening, COPD- Chronic obstructive pulmonary disease, CKD- Chronic kidney disease, DM- Diabetes mellitus, ICU- Intensive care unit

NRS Score	Grade	Impaired nutritional status	Disease severity according to metabolism or requirement
1	Mild	Weight loss> 5% in last three months Or Food intake < 50-75% of normal diet requirement in the preceding week	Hip fracture Chronic disease with acute complications like COPD, Cirrhosis, CKD on hemodialysis, DM, malignant oncology
2	Moderate	Weight loss> 5% in last two months Or Food intake < 25-50% of normal diet requirement in the preceding week Or BMI 18.5-20.5 with impaired general condition	Major abdominal surgery Stroke Severe pneumonia Malignant hematology
3	Sever	Weight loss> 5% in last one month/ > 15 in last three months Or Food intake < 0-25% of normal diet requirement in the preceding week Or BMI <18.5 with impaired general condition	Head injury Bone marrow transplantation ICU patients(Apache>10)
Total score	Impaired nutritional status score plus		Impaired nutritional status score
Note:	Age>70 – add 1 into a total score Total score 0- Normal nutritional status or requirements Total score 3 or > 3- The patient is nutritionally at risk and nutritional care should be initiated Total score <3- weekly basis nutritional screening should be planned		

The postoperative anasarca refers to generalized edema following surgery, whether due to underlying preoperative conditions, inadvertent intravenous fluid management or perioperative complications. Anasarca can manifest itself as pitting edema of the extremities with the face, chest, or abdomen. Patients with this condition may have poor recovery in the early postoperative period and have long hospital stays. Leukocytosis is a hematological condition that is characterized by an increase in the number of white blood cells (WBC > 11000/mm3) in the blood circulation.

The patient outcomes like mobilization, ability to tolerate semisolids, time to bowel movement were measured in the postoperative period. The requirement of supplemental products like blood, TPN, and albumin was documented. Postoperative complications were evaluated by the Clavien-Dindo grading system. Complications like skin dehiscence, fascial dehiscence, leak of repaired perforation or anastomosis, reoperation, readmissions, ICU admissions and mortality were recorded.

The data collected during the study were entered into an excel sheet. The data were tabulated and analyzed using Statistical Package for Social Sciences version 25 (IBM SPSS Statistics v25, IBM Corp., Armonk, NY, USA). The incidence of anasarca was calculated. The percentages, mean and standard deviation of the parameters were calculated. The parameters like age groups, NRS 2002 score, BMI, smoking, alcohol, opium intake, and leucocytosis were assessed by the Pearson Chi-square test. The parameters like hemoglobin, mid-arm circumference, mid-thigh circumference, and albumin were assessed by Student’s T-test. The parameter Charlson comorbidity index was evaluated by the Mann-Whitney U test. The p-value <0.05 is considered statistically significant.

Ethical clearance was obtained from the Institutional Ethics Committee at All India Institute of Medical Sciences, Jodhpur (AIIMS/IEC/2019-20/844). All patients were enrolled after taking informed consent. Patient details were kept confidential.

## Results

**Figure 1:**
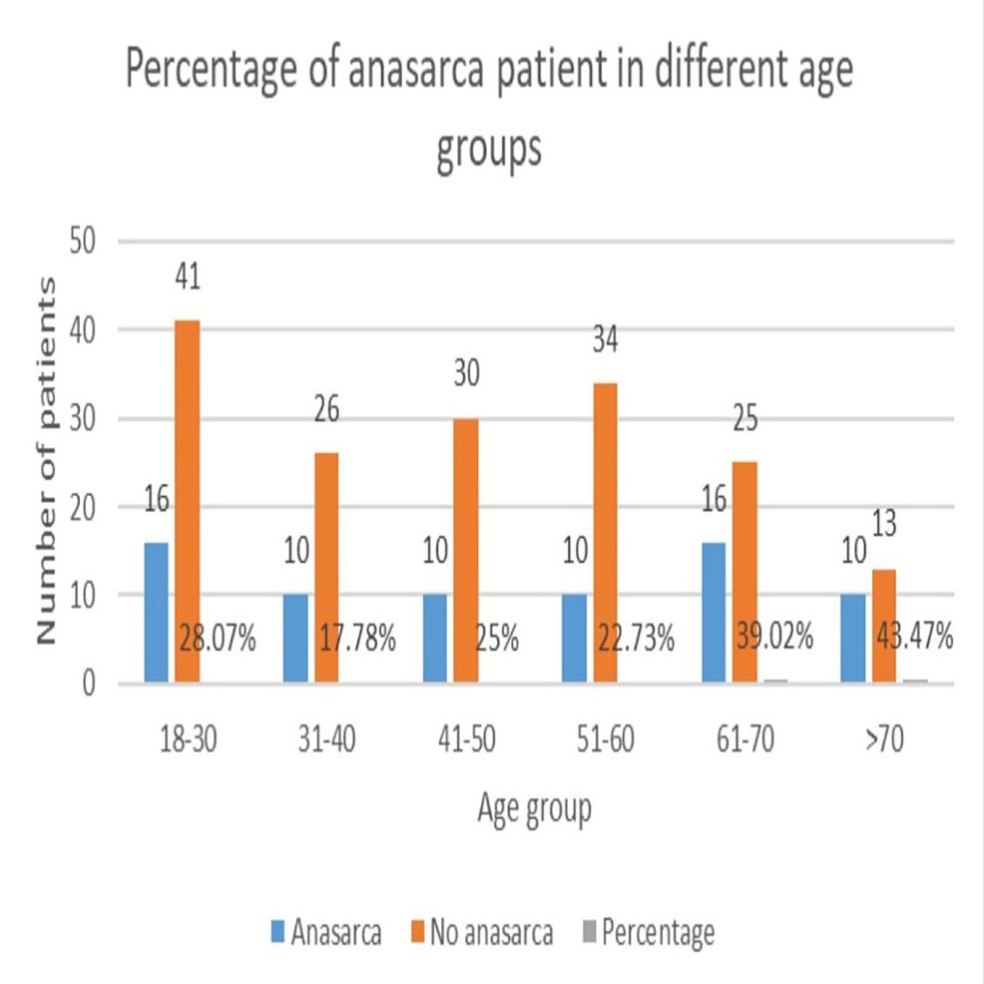
Demographic profile of study participants.

In this prospective observational study, 241 patients were recruited who underwent major abdominal surgery and 72 (29.87%) patients had developed postoperative anasarca. The mean (SD) age for the 72 patients who developed anasarca was 50.93(4.60) years. Pearson Chi-square test was implemented and the age > 60 years was found to be statistically significant (p < 0.05) (Figure 1).

Out of 241 patients, 152 were male and 89 were female. A total of 16.60% of males and 13.28% of the females developed anasarca post-operatively. In the study, 106 (43.98%) patients had NRS ≥ 3, which indicates that they are nutritionally at risk. The NRS value was found to be statistically significant (p < 0.05). 

The p-value (>0.05) was not significant for mid-arm circumference, mid-thigh circumference, and body mass index. A total of 14 out of 72 patients developing anasarca had BMI < 18.5 kg/m2 indicating underweight or malnutrition, whereas 58 out of 169 patients who did not develop anasarca had a BMI of <18.5 kg/m2. Therefore, lower BMI values are not associated with a higher incidence of postoperative anasarca. The p-value (<0.05) for albumin was significant and indicates that lower albumin levels were associated with an increased chance of development of postoperative anasarca. Hemoglobin and CCI were not significantly different between the patients who developed anasarca and those that did not develop anasarca (Table 2). A total of 46 (63.9%) patients associated with anasarca and 66 (23%) patients without anasarca had been found to have leukocytosis (>11000/mm3) preoperatively. The p-value was statistically significant (p <0.05). 

**Table 2:** Risk factors associated with postoperative anasarca patients NRS-Nutritional risk screening score, MAC- Mid-arm circumference, MTC- Mid-thigh circumference, BMI- Body mass index, CCI- Charlson-comorbidity index

Risk Factors	Anasarca ( Mean±SD)	Without Anasarca (Mean±SD)	P-value
Age	50.93 ± 4.60	46.53 ± 2.62	0.027
NRS	2.98 ± 0.36	2.08 ± 0.21	0.001
MAC	23.83 ± 0.81	24.30 ± 0.52	0.347
MTC	42.78 ± 1.40	42.40 ± 0.82	0.637
BMI	21.91 ± 0.90	21.93 ± 0.62	0.526
Hemoglobin	11.24 ± 0.61	11.88 ± 0.32	0.051
Leukocytosis	14.39 ± 2.14	11.06 ±1.02	0.001
Albumin	2.64 ± 0.16	3.47 ± 0.12	0.001
CCI	1.99 ± 0.52	1.86 ± 0.31	0.808

In this study, 45 (62.5%) patients with anasarca had severe complications according to Clavien-Dindo grading IV or V (p-value<0.001) (Table 3).

**Table 3:** Clavien-Dindo Grading of complications following major abdominal surgery n- Patient number, no- Number

Clavien-Dindo grade	Anasarca (n=72)	Without anasarca (n=169)	P-value
	Patients no (Percentage)	Patients no (Percentage)	
Grade 1	12(16.7%)	61(14.8%)	0.002
Grade 2	10(13.9%)	14(8.3%)	0.199
Grade 3	1(1.4%)	13(7.7%)	0.049
Grade 4	15(20.8%)	9(5.3%)	<0.001
Grade 5	30(41.7%)	9(5.3%)	<0.001

A total of 23, 11 and 12 patients with postoperative anasarca were habituated to smoking, alcohol and opium respectively, while 40, 21 and 16 patients without anasarca were habituated for the same. The p-value for these variables was not found to be statistically significant (p > 0.05). The average time to mobilization, initiation of semisolid and bowel movement in the surviving patients was 2.6, 4.3 and 4.6 days respectively. Blood, total parenteral nutrition, albumin had to be administered to 51%, 55% and 26% of anasarca patients due to low levels of hemoglobin and albumin postoperatively. 

A total of 40 anasarca patients had required ICU admission postoperatively for intensive care and monitoring or ventilator support (p value<0.001). A total of 18 anasarca patients had to be taken up for surgery for various reasons following primary surgery (p-value=0.027). More than 60% of anasarca patients had skin dehiscence, 20% had fascial dehiscence and 35% had an intestinal leak (p value<0.05) (Table 4).

**Table 4:** Postoperative outcome among all study participants n- Patient number, no- number, ICU- Intensive care unit

Patient outcome	Anasarca (n=72)	Without anasarca (n=169)	P- value
	Patients no(Percentage)	Patients no(Percentage)	
Skin dehiscence	46 (63.9%)	40(23.7%)	<0.001
Fascial dehiscence	14 (19.4%)	4 (2.4%)	<0.001
Intestinal leak	8/23 (34.8%)	9/76 (11.8%)	0.019
ICU admission	40 (55.5%)	16 (9.5%)	<0.001
Revision surgery	18 (25%)	22 (13.0%)	0.027
Mortality	30 (41.67%)	9 (5.33%)	<0.001
Readmission among survival patients	5/42 (11.90%)	19/167 (11.38%)	0.896

## Discussion

Postoperative anasarca is a significant factor affecting patient outcomes. In this prospective observational study of 241 patients, almost 30% of patients developed postoperative anasarca. Vaughan et al. conducted a study on 55 patients undergoing major abdominal surgery in which 19 had developed anasarca postoperatively, which amounted to 34.55% [3]. Postoperative anasarca was most prevalent, and also significantly associated with age groups greater than 60 years. Claudia et al. conducted a study on 308 patients undergoing abdominal surgery for cancer and concluded that the group of patients who developed complications was older (63.3 ± 12.5 years) [4]. Massarweh et al. reported a study in 318 patients of 65 years or older and found out that the frequency of 90-day postoperative complications increased with each five-year incremental increase in age (65-69 years, 14.6%; 70-74 years, 16.1%; 75-79 years, 18.8%; 80-84 years, 19.9%; 85-89 years, 22.6%; and ≥90 years, 22.7%; trend test, p-value < .001) [5]. Postoperative anasarca following abdominal surgery may be more prevalent in elderly age groups because the physiological safety mechanisms which protect against the formation of edema deteriorate with age.

The NRS 2002 score was developed and validated to assess the risk of malnutrition in hospitalized patients and is recommended by the American College of Gastroenterology guidelines. NRS 2002 score of ≥ 3 indicates that the patient is nutritionally at risk. Higher NRS scores were associated with a higher incidence of development of anasarca postoperatively. These results are comparable to the results of a study done by Zhen et al., who concluded that patients with preoperative nutritional risk have increased complication rates following surgery. Acute malnutrition may lead to physiological derangements of body and increased incidence of edema [6].

Hemoglobin levels were not found to be statistically significant for the incidence of postoperative anasarca but the p-value was borderline (0.051). Miles et al. correlated borderline anemia to postoperative complications and found uncertainty in their relationship [7]. Preoperative moderate-severe anemia resulted in a fourfold increase in the rate of postoperative complications in patients undergoing laparotomies according to Sincavage et al. [8].

Lower preoperative albumin levels were significantly associated with a higher incidence of postoperative anasarca following laparotomies. In the study conducted by Vaughan et al., the average value of albumin levels for those who developed anasarca was 2.20 gm/dL, and for those who didn’t develop anasarca was 2.47 gm/dL as compared to 2.64 gm/dL and 3.47 gm/dL in our study. The study has found a significant correlation between the level of preoperative albumin and the development of anasarca postoperatively, whereas Vaughan et al. found no significant correlation. Gibbs et al. suggested that albumin reduction from 4.6 g/dL to <2.1 g/dL was associated with increased mortality and morbidity, especially in sepsis and major infections [3,9]. Ryan et al. had done a retrospective study on 200 patients who underwent esophagectomy for malignancy and found that albumin on the first postoperative day, was a superior predictor of surgical outcomes compared to various other preoperative parameters [10].

The average fall in albumin in the patients who developed anasarca from preoperative value to that on the first postoperative day is 0.83 gm/dL. Labgaa et al.'s study concluded that a fall of ≥ 1.0 gm/dL was associated with a threefold increase in complications postoperatively [11]. Albumin is perhaps a major factor in the maintenance of plasma colloid osmotic pressure, and thereby its levels are directly correlated with the development of anasarca. Anthropometric measurements like MAC, MTC, and BMI were not found to be significantly correlated to the development of anasarca postoperatively. Vaughan et al. also had the same findings in their study that BMI is not associated with postoperative anasarca. Bharathi et al. conducted a study in 130 patients and came to the result that MAC was not significantly correlated with postoperative complications [3,12]. Thereby anthropometry may not be a reliable predictor of the development of postoperative anasarca.

According to our study, addictions like smoking, tobacco, alcohol, or opium intake did not significantly correlate to the development of anasarca postoperatively. Inoue et al. observed that the length of hospital stay was longer and the suture failure rate was greater in smokers. [13]. Dahl et al. also suggested that smoking and alcohol abuse were significantly correlated with wound-related complications and death but they did not correlate it with postoperative edema [14].

Preoperative raised leukocyte counts (>11,000 cells/mm³) can be considered a marker for sepsis and directly correlate with the development of anasarca postoperatively. Moghadamyeghaneh et al. conducted a retrospective study on 59,805 patients undergoing surgical resection for colorectal cancer from the NSQIP database and concluded that preoperative asymptomatic leukocytosis had significant associations with increased mortality and morbidity of patients [15]. Our findings may be explained because increased leukocyte counts are due to the presence of more inflammation in the body and are also proportional to the number of inflammatory mediators circulating in the body. These inflammatory mediators are responsible for vasodilation and increased permeability leading to the development of edema and anasarca.

Postoperative complications of patients who developed anasarca postoperatively were graded as per the Clavien-Dindo grading system. The postoperative outcome in terms of complications was significant in grades 4, 5 (p value<0.001). The low number of patients in grade III may be attributed to the fact that most of the patients who needed surgical or radiological intervention following primary surgery were shifted to the intensive care unit following physiological deterioration, and thereby their complication grade shifted upwards. Few studies have shown increased rates of complications in patients developing edema [3,15,16].

Charlson comorbidity index (CCI) was calculated for the subjects and was correlated to the development of postoperative anasarca. It was not found to be statistically significant. Strombom et al. concluded that in patients undergoing colorectal surgery, comorbidity indices do not risk adjusting for surgical complications, surgical site infections, or Clavien Dindo complications of grade 3 or above, anastomotic leak and abscess [16]. However, a retrospective study by Carmen et al. suggested that CCI has significant importance in predicting postoperative outcomes [17].

The period of mobilization differs according to the type of surgery performed. It also depends on other accompanying factors like limb injury in trauma patients, deep vein thrombosis, osteoarthritis, debilitating condition, and requirement of ventilator support in ICU following surgery. The average period to intake of semisolids is dependent on multiple factors like an indication of surgery and type of surgery done (for example, anastomosis versus stoma, site of operation like upper and lower gastrointestinal tract sites, emergency vs elective surgery). Morgan et al. concluded that the role of early oral feeding is yet uncertain in case of emergency surgeries [18]. The average period from surgery to bowel movement also depends upon the age, indication and duration of surgery performed, presence of Ryle's tube, period to initiation of mobilization, presence of abdominal drains and intraoperative bowel condition. The edematous bowel may have increased postoperative ileus [19].

In this study, the requirement of administration of supplemental products like blood, total parenteral nutrition (TPN), and albumin was increasing in postoperative anasarca patients. Albumin supplementation does not have much of a role as a nutritional supplement as per the literature review [20]. Still, albumin does have a role in septic shock to increase the intravascular fluid volume and maintain adequate circulation in the body. More than 50% of patients had ICU admissions and about one-third of total intestinal anastomosis or repairs had suture leaks in postoperative anasarca patients. Previous literature suggested leak rates of 0.9 - 3.5% after bowel anastomosis [21]. A total of 25% of patients had required reoperation, either early or delayed, for various complications following primary surgery. More than 60% of patients had dehiscence of skin and nearby 20% had fascial dehiscence in our study. Aga et al. obtained a surgical site infection rate of one-third of our study. Vaughan et al. obtained mortality results of 47% and comparable with our results in patients who had postoperative anasarca [3,22].

The strength of this study was that this was a prospective study, which included major abdominal surgery and had a large sample size. This study also has a few limitations. The cohort included patients undergoing laparotomy for many different indications like bowel obstruction, intestinal perforation, peritonitis due to pancreatic-biliary cause, an intraperitoneal collection due to infection or traumatic cause with very different preoperative nutritional characteristics. The postoperative outcome like mobilization, oral intake may be affected by confounding factors like type and duration of surgery and pain control strategies like epidural analgesia, patient-controlled analgesia, local nerve blocks. Patients having stoma or non-bowel abdominal surgery initiated on feeds earlier than those having bowel anastomosis done, and period to bowel movement also varies accordingly.

## Conclusions

A higher NRS 2002 score, lower albumin levels, higher age >60 years, and a higher leukocyte count are significantly correlated with the development of postoperative anasarca following major abdominal surgery. Anasarca after surgery is associated with a poor prognosis in these patients. The majority of these patients may have higher Clavien-Dindo grades and require intensive care monitoring. Postoperative complications like skin dehiscence, fascial dehiscence, and multiple organ dysfunction as well as mortality are increased in postoperative anasarca patients. Postoperative anasarca can lead to long hospital stays and delayed postoperative recovery. Appropriate identification of high-risk factors and preoperative planning can reduce the severity of postoperative morbidity and mortality.

## References

[1] Eknoyan G (1997). A history of edema and its management. Kidney Int Suppl.

[2] Guo D, Gong J, Cao L, Wei Y, Guo Z, Zhu W (2016). Laparoscopic surgery can reduce postoperative edema compared with open surgery. Gastroenterol Res Pract.

[3] Vaughan-Shaw PG, Saunders J, Smith T, King AT, Stroud MA (2013). Oedema is associated with clinical outcome following emergency abdominal surgery. Ann R Coll Surg Engl.

[4] Simões CM, Carmona MJ, Hajjar LA (2018). Predictors of major complications after elective abdominal surgery in cancer patients. BMC Anesthesiol.

[5] Massarweh NN, Legner VJ, Symons RG, McCormick WC, Flum DR (2009). Impact of advancing age on abdominal surgical outcomes. Arch Surg.

[6] Sun Z, Kong XJ, Jing X, Deng RJ, Tian ZB (2015). Nutritional risk screening 2002 as a predictor of postoperative outcomes in patients undergoing abdominal surgery: a systematic review and meta-analysis of prospective cohort studies. PLoS One.

[7] Miles LF, Larsen T, Bailey MJ, Burbury KL, Story DA, Bellomo R (2020). Borderline anaemia and postoperative outcome in women undergoing major abdominal surgery: a retrospective cohort study. Anaesthesia.

[8] Sincavage J, Robinson B, Msosa VJ, Katete C, Purcell LN, Charles A (2021). Preoperative anemia and surgical outcomes following laparotomy in a resource-limited setting. Am J Surg.

[9] Gibbs J, Cull W, Henderson W, Daley J, Hur K, Khuri SF (1999). Preoperative serum albumin level as a predictor of operative mortality and morbidity: results from the National VA Surgical Risk Study. Arch Surg.

[10] Ryan AM, Hearty A, Prichard RS, Cunningham A, Rowley SP, Reynolds JV (2007). Association of hypoalbuminemia on the first postoperative day and complications following esophagectomy. J Gastrointest Surg.

[11] Labgaa I, Joliat GR, Kefleyesus AA (2016). Postoperative decrease of serum albumin is an early predictor of complications after major abdominal surgery: a prospective cohort study. J Am Coll Surg.

[12] Akula B, Nagral S, Nilesh Nilesh (2019). Impact of nutrition on complications after abdominal surgery. Adv Surg Res.

[13] Inoue Y, Katoh T, Masuda S (2020). Perioperative complications of abdominal surgery in smokers. J Anesth.

[14] Dahl RM, Wetterslev J, Jorgensen LN, Rasmussen LS, Moller AM, Meyhoff CS (2014). The association of perioperative dexamethasone, smoking and alcohol abuse with wound complications after laparotomy. Acta Anaesthesiol Scand.

[15] Moghadamyeghaneh Z, Hanna MH, Carmichael JC, Mills SD, Pigazzi A, Stamos MJ (2015). Preoperative leukocytosis in colorectal cancer patients. J Am Coll Surg.

[16] Strombom P, Widmar M, Keskin M (2019). Assessment of the value of comorbidity indices for risk adjustment in colorectal surgery patients. Ann Surg Oncol.

[17] Payá-Llorente C, Martínez-López E, Sebastián-Tomás JC, Santarrufina-Martínez S, de'Angelis N, Martínez-Pérez A (2020). The impact of age and comorbidity on the postoperative outcomes after emergency surgical management of complicated intra-abdominal infections. Sci Rep.

[18] Le Guen M, Fessler J, Fischler M (2014). Early oral feeding after emergency abdominal operations: another paradigm to be broken?. Curr Opin Clin Nutr Metab Care.

[19] Pozios I, Seeliger H, Lauscher JC (2021). Risk factors for upper and lower type prolonged postoperative ileus following surgery for Crohn's disease. Int J Colorectal Dis.

[20] Mahkovic-Hergouth K, Kompan L (2011). Is replacement of albumin in major abdominal surgery useful?. J Clin Anesth.

[21] Hyman N, Manchester TL, Osler T, Burns B, Cataldo PA (2007). Anastomotic leaks after intestinal anastomosis: it's later than you think. Ann Surg.

[22] Aga E, Keinan-Boker L, Eithan A, Mais T, Rabinovich A, Nassar F (2015). Surgical site infections after abdominal surgery: incidence and risk factors. A prospective cohort study. Infect Dis (Lond).

